# Associations between missing teeth and the risk of cancer in Korea: a nationwide cohort study

**DOI:** 10.1186/s12903-023-02997-x

**Published:** 2023-06-23

**Authors:** Eun Joo Kang, Seok-joo Moon, Kyoungmin Lee, In Hae Park, Jung Sun Kim, Yoon Ji Choi

**Affiliations:** 1 Department of Internal Medicine, Korea University Guro Hospital, Korea University College of Medicine, 148, Gurodong-Ro, Guro-Gu, Seoul, 08308 Republic of Korea; 2 Smart Healthcare Center, Korea University Guro Hospital, 148, Gurodong-Ro, Guro-Gu, Seoul, 08308 Republic of Korea; 3 Department of Internal Medicine, Korea University Ansan Hospital, Korea University College of Medicine, 123, Jeokgeum-Ro, Danwon-Gu, Ansan, Gyeonggi-Do 15355 Republic of Korea; 4 Department of Internal Medicine, Korea University Anam Hospital, Korea University College of Medicine, 73 Goryeodae-Ro Seongbuk-Gu, Seoul, 02841 Republic of Korea

**Keywords:** Tooth loss, Oral health, Neoplasms, Risk

## Abstract

**Background:**

Poor dental health is correlated with an increased risk of cancer. Using a nationwide population cohort database, we investigated which cancer is highly associated with poor dental health and which dental indicator mostly influences cancer risk.

**Methods:**

This study was conducted using the National Health Checkups (NHC) and National Health Insurance System (NHIS) database in Korea. NHC in Korea includes dental examinations. We retrieved subjects who underwent NHC between 2002 and 2003 and their medical information in NHIS database was followed until December 31,2015.

**Results:**

Data for 200,170 who participated in the NHC between 2002 and 2003 were analysed. During the maximum follow-up period of 13 years, 15,506 (7.75%) subjects were diagnosed with cancer. The median time to cancer diagnosis after the dental examination was 87 months (range, 51–119 months). The proportion of people with missing teeth was higher in the cancer-diagnosed group than in the non-diagnosed group (26.27% vs. 22.59%, p < 0.001). Among several dental health factors, missing teeth were significantly associated with higher cancer risk. Subjects with missing teeth showed a 12% increased cancer risk compared to those without missing teeth (odds ratio [OR] 1.12, 95% confidence interval [CI], 1.08–1.16). The risk was significantly higher, especially in lung, head and neck, pancreatic, liver, biliary, and esophageal cancers (OR 1.27 [95% CI, 1.14–1.41], 1.32 [95% CI, 1.13–1.55], 1.27 [95% CI, 1.02–1.58], 1.24 [95% CI, 1.1–1.4], 1.28 [95% CI, 1.03–1.6], 1.4 [95% CI, 1.04–1.88], respectively).

**Conclusions:**

Missing teeth were the most important dental indicator associated with cancer risk. Korean adults with missing teeth should be cautious about the risk of several cancers, particularly head and neck, lung, gastrointestinal, hepatobiliary, and pancreatic cancer.

## Introduction

Dental health is correlated with several systemic diseases, such as rheumatologic disease, cardiovascular disease, diabetes, and cancers [1–6]. Dental caries, the number of missing teeth, and periodontitis are widely used as representative indicators of dental health. According to the statistics from the Korea Centers for Disease Control and Prevention, the prevalence of dental caries and periodontal disease for permanent teeth among those aged 19 years and over was 29.1% and 23.4% of each in 2016–2018. Moreover, 54.3% of the population who conducted the Korea National Health and Nutrition Examination Survey from 2010–2015 reported having at least one missing tooth [7, 8]. Periodontitis is a common chronic inflammatory condition caused by intraoral bacterial microorganisms, which gradually destroy periodontal soft tissue and eventually affect the teeth [9]. Dental caries results from complex processes induced by intraoral bacteria, fermentable carbohydrates, and other host factors on teeth. Most importantly, missing teeth in adulthood are regarded as the final result of seriously progressed inflammatory conditions in periodontal soft tissue and teeth. Several reports suggest that these intraoral microbiota compositions and inflammatory reactions are associated with systemic conditions [10]. In addition, some cancers are directly related to chronic inflammatory conditions in their pathogenesis [11]. Besides well-known oncogenic viruses like human papillomavirus, Epstein-Barr virus, and Hepatitis B virus, intraoral microbiota have also been identified as highly associated with increased risk of various diseases, including cancer, by creating a pro-inflammatory microenvironment and impairing the immune response [12, 13].

In Korea, dental health screening is included in the National Health Checkups (NHC) program provided by the government for all populations in Korea. Therefore, dentists regularly check their dental health status. Based on data from the NHC and engaged health insurance claims data of this population, we investigated which cancer is highly associated with poor dental status and which dental factor can be the most reliable index associated with the risk of cancer incidence among the Korean population.

## Materials and methods

### Data source

This study was conducted using NHC database of the National Health Insurance Service (NHIS) in Korea. Because nearly 98% of citizens who reside in Korea, except for Medical Aid beneficiaries and Health Care beneficiaries for veterans, are assigned to the NHIS program.

As part of the NHIS system, all insurance subscribers and dependents are asked to take a free biannual NHC. The NHC comprised a general health examination and a dental health examination. The NHIS has been operating the National Health Insurance Sharing Service (NHISS) and provides national health information including NHC data to Korean researchers to conduct policy and academic researches upon approval [14]. Database also includes insurance rate, medical check-up result, treatment details, elderly long-term nursing insurance data, clinic status, registered information of cancer and rare disease, and etc.

This study was conducted in accordance with the Declaration of Helsinki, Good Clinical Practice guidelines, and national policy on the Personal Information Protection Act. This study was approved by the Institutional Review Board (IRB) of the Korea University Guro Hospital (IRB No. 2020GR0173) and accepted by the NHIS (NHIS-2022–2-037). Consent to participate in the research was waived by IRB of Korea University Guro Hospital because we used anonymized data for the retrospective analysis.

### Study population and definitions

We retrieved the records of 514,886 people who participated in the NHC program between January 1, 2002, and December 31, 2003. Afterward, newly onset medical information after the NHC were followed until December 31, 2015. To obtain information on newly diagnosed cancer, we retrieved the data of individuals with diagnosis codes on the International Statistical Classification of Diseases and related health problems 10th revision (ICD-10) and additional V193 code for cancer patients. The V193 code is specifically used for all cancer patients in Korea to support medical expenses for cancer diagnosis and treatment. Therefore, all claims applied to the NHIS of patients with cancer have additional V193 code. Also, for the exact definition of newly diagnosed patients during this time, we applied a 1-year washout window period before detecting the ICD-10 code for cancer. Thus, individuals assigned an ICD-10 code for cancer in 2002 were not considered new patients. Individuals who had cancer codes between January 1, 2002, and December 31, 2002, and who already had cancer codes in medical claims before NHIS were excluded from the analysis. Although all claims data provided by NHIS conceal individuals’ identities according to the Act on the Protection of Personal Information Maintained by Public Agencies, claim data of certain cancers, including breast cancer, genital tract cancers, and prostate cancer, which were designated as sensitive diagnoses for privacy by NHIS, were not provided without additional requests; therefore, we excluded data of these cancers in the analysis.

A dentist performed dental examinations for NHC at the institution assigned by the government. Only approved dentists who completed online training program for NHC could participate in NHC. In addition to the training program, the Korea Centers for Disease Control (KCDC) and the NHIS provided a guideline for recording NHC results for reliability among the examiners [15]. In dental examinations, information about the presence of dental caries and their number, periodontal disease, and status of dental loss was obtained. The examiners used the same definitions for diagnosing dental caries or periodontitis according to the guideline. In the case of tooth loss, only the case by dental caries or inflammatory causes without trauma, injury, artificial tooth or other orthodontic reasons had to be defined as tooth loss in NHC. In addition, smoking status, alcohol consumption, body mass index (BMI), underlying diseases such as diabetes mellitus, hypertension, cardiac disease, cerebrovascular disease, and economic status were obtained from the NHIS.

### Statistical analysis

All subjects with missing data were excluded from the statistical analysis. Chi-square analysis was performed to compare categorical variables, and Student’s t-test was performed to compare continuous variables between the groups with and without cancer. A multivariable logistic regression model was used to analyze the risk factors for cancer occurrence, adjusting for age, sex, income, smoking, alcohol consumption, and comorbidities. Kaplan –Meier survival curves were used to analyze the cumulative incidence of cancer. Statistical significance was set at p < 0.05. All statistical analyses were performed using SAS software (version 9.4; SAS Institute Inc.) and R statistical software, version 3.3.3 (R Foundation Inc.; http://cran.r-project.org/).

## Results

**Fig. 1:**
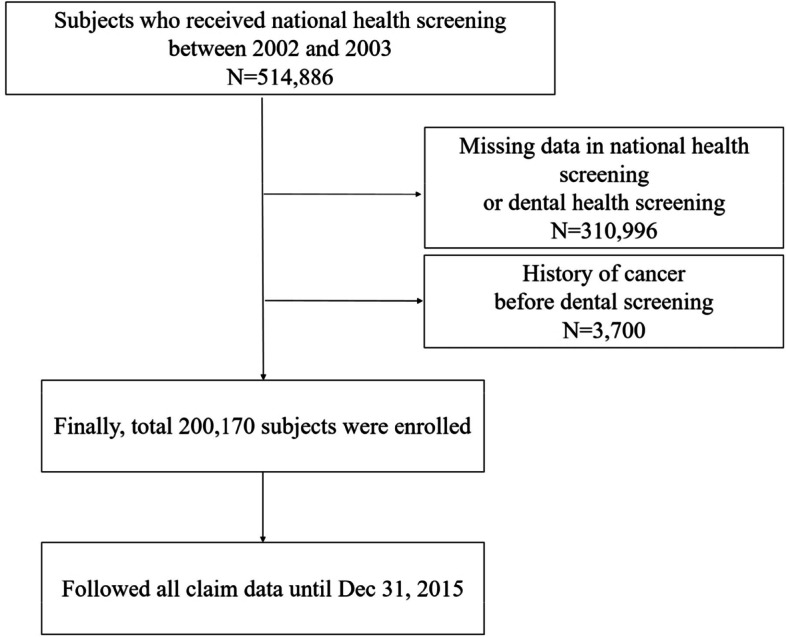
Flow chart of the study subjects

From data from 514,886 people who underwent national health examinations between December 31, 2002, and December 31, 2003, data from 310,996 people were excluded for missing components or absence of dental examination. In addition, data from 3,700 people were excluded for previous cancers before the dental examination. Eventually, all claims data from 200,170 were followed until December 31, 2015 (Fig. 1).

The baseline characteristics of all included individuals are listed in Table 1. The included individuals were aged from 40 to 79 years, with a median age of 50.8 years. Most participants were aged less than 65 years (90.3%), and males comprised 61.6% of the total population. The proportion of people with a smoking history was 37.5% of the total population, and 10.9% consumed alcohol more than 10 times monthly. We evaluated economic status by the amount of payment for the national health insurance because the government decided the payment according to the income. Less than the third quantile, people with low economic status comprised 20.7% of the total population. Regarding comorbid diseases, 34.4% were obese with a BMI ≥ 25. Also, 15.8% of the study population had hypertension, and 6.5% had diabetes.

**Table 1:** Baseline characteristics of the study population

	Total participants *n* = 200,170	
Median age, y	50.82	
Age group	N	%
< 65	180,704	90.28
≥ 65	19,466	9.72
Sex		
Male	123,332	61.61
Female	76,838	38.39
Income		
≤ Third quantiles	41,410	20.69
> Third quantiles	158,760	79.31
Smoking		
Never	125,026	62.46
Ex-smoker	21,808	10.89
Current smoker	53,336	26.65
Alcohol consumption		
< 10 times per month	178,283	89.07
≥ 10 times per month	21,887	10.93
Dental health status		
Number of dental caries		
0	184,555	92.20
1- 4	12,416	6.20
≥ 5	3,199	1.60
Missing teeth	45,796	22.88
Periodontitis	101,796	50.5
Comorbidity		
Obesity	68,879	34.41
Hypertension	31,694	15.83
Diabetes	13,021	6.50
Heart disease	5,571	2.78
Cerebrovascular disease	3,398	1.70
Cancers		
All cancers^a^	15,506	7.75
Gastric cancer	3,223	1.61
Colorectal cancer	2,461	1.23
Thyroid cancer	2,222	1.11
Lung cancer	1,797	0.90
Liver cancer	1,428	0.71
Head and neck cancer	740	0.37
Hematologic malignancies	550	0.27
Bladder cancer	499	0.25
Kidney cancer	417	0.21
Pancreatic cancer	409	0.20
Gallbladder / biliary tract cancer	407	0.20
Esophageal cancer	207	0.10
Other cancers	1,146	0.57

^a^Information on breast cancer, female and male genital tract cancers and prostate cancer are not included in this analysis

For dental health status, we focused on three major dental indicators: dental caries, missing teeth, and periodontitis, because they can be regarded as objectively described by dentists. Among the total population, 7.8% had untreated dental caries, and 22.9% had more than one missing tooth. Approximately half (50.9%) of the subjects had periodontitis. In total, 15,506 (7.6%) subjects were diagnosed with cancer after the NHSE. Gastric cancer was the most commonly diagnosed cancer, followed by colorectal cancer, thyroid cancer, lung cancer, liver cancer, and head and neck cancer. Among the 45,796 subjects with missing teeth, 4,074 subjects (8.9%) were diagnosed with cancer after the dental examination.

We performed a chi-square test to analyze the characteristics of patients diagnosed with cancer or not (Table 2). The proportion of elderly (≥ 65 years old), male, people with low economic status, smoking history, frequent alcohol consumption, and comorbid diseases was higher in people diagnosed with cancer. The proportion of people without dental caries was slightly higher in the cancer group, and the proportion of patients with periodontitis was not different between the cancer and non-cancer groups. However, the proportion of people with missing teeth was higher in the cancer group (26.27% vs. 22.59%, *p* < 0.001).

**Table 2:** Comparison of characteristics between cancer—undiagnosed group and cancer—diagnosed group

	Cancer – undiagnosed group *N* = 184,664		Cancer – diagnosed group *N* = 15,506		*p*-value
Median age	50.51		54.58		< .0001 (a)
Age group, y					< .0001 (b)
< 65	167,964	(90.96)	12,740	(82.16)	
≥ 65	16,700	(9.04)	2,766	(17.84)	
Sex					< .0001 (b)
Male	112,842	(61.11)	10,490	(67.65)	
Female	71,822	(38.89)	5,016	(32.35)	
Income					< .0001 (b)
≤ Third quantiles	37,973	(20.56)	3,437	(22.17)	
> Third quantiles	146,691	(79.44)	12,069	(77.83)	
Smoking					< .0001 (b)
Never	115,998	(62.82)	9,028	(58.22)	
Ex-smoker	20,011	(10.84)	1,797	(11.59)	
Current smoker	48,655	(26.35)	4,681	(30.19)	
Alcohol consumption					< .0001 (b)
< 10 times per month	165,045	(89.38)	13,238	(85.37)	
≥ 10 times per month	19,619	(10.62)	2,268	(14.63)	
Dental health status					
Number of dental caries					0.0179 (b)
0	170,177	(92.15)	14,378	(92.73)	
1–4	11,536	(6.25)	880	(5.68)	
≥ 5	2,951	(1.60)	248	(1.60)	
Missing teeth	41,722	(22.59)	4,074	(26.27)	< .0001 (b)
Periodontitis	93,821	(50.81)	7,975	(51.43)	0.1346 (b)
Comorbidity					
Obesity	63,391	(34.33)	5,488	(35.39)	0.0073 (b)
Hypertension	28,550	(15.46)	3,144	(20.28)	< .0001 (b)
Diabetes	11,637	(6.30)	1,384	(8.93)	< .0001 (b)
Heart disease	4,990	(2.70)	581	(3.75)	< .0001 (b)
Cerebrovascular disease	3,065	(1.66)	333	(2.15)	< .0001 (b)

(a) *p*-value by Students t-test; (b) *p*-value by the chi-square test

Multivariate logistic regression analysis was performed to analyze risk factors for cancer (Table 3). In a multivariable logistic regression model adjusted for age, sex, income, smoking, alcohol consumption, and comorbidities, older age (> 65 years), male sex, low economic status, smoking history, and frequent alcohol consumption were found to be influential factors related to increased cancer risk. In addition, comorbid diseases, including hypertension, diabetes, and heart diseases, were also associated with increased cancer risk.

**Table 3:** Multivariate analysis for risk for several cancers

	All cancers			Gastric Cancer			Lung cancer			Liver Cancer			Colorectal cancer		
	OR	95% CI	*P*	OR	95% CI	*P*	OR	95% CI	*P*	OR	95% CI	*P*	OR	95% CI	*P*
Age group, y			<.0001			<.0001			<.0001			<.0001			<.0001
<65	Ref.			Ref.			Ref.			Ref.			Ref.		
≥65	2.11	2.01-2.21		2.40	2.18-2.65		3.74	3.33-4.19		1.89	1.62-2.21		2.14	1.92-2.39	
Sex			<.0001			<.0001			<.0001			<.0001			<.0001
Male	Ref.			Ref.			Ref.			Ref.			Ref.		
Female	0.75	0.72-0.78		0.43	0.39-0.48		0.55	0.48-0.63		0.29	0.25-0.35		0.57	0.52-0.64	
Income						0.0209			<.0001			<.0001			0.0128
≤Third quantiles	Ref.		<.0001	Ref.			Ref.			Ref.			Ref.		
>Third quantiles	0.89	0.86-0.93		0.90	0.82-0.98		0.80	0.72-0.9		0.75	0.66-0.86		0.88	0.8-0.97	
Smoking			<.0001			0.1531			<.0001			0.4316			0.2442
Never	Ref.			Ref.			Ref.			Ref.			Ref.		
Ex-smoker	1.03	0.97-1.09		1.06	0.95-1.19		1.16	0.98-1.38		1.09	0.93-1.29		1.11	0.98-.127	
Current smoker	1.10	1.06-1.15		1.09	1-1.19		2.04	1.82-2.3		1.07	0.95-1.22		1.02	0.92-1.13	
Alcohol consumption			<.0001			<.0001			<.0001			0.0014			0.0060
<10 times per month	Ref.			Ref.			Ref.			Ref.			Ref.		
≥10 times per month	1.26	1.20-1.33		1.32	1.2-1.45		1.34	1.19-1.52		1.26	1.09-1.45		1.18	1.05-1.33	
Dental health status															
Dental caries			0.009			0.0945			0.4393		0.3282				0.0343
0	Ref.			Ref.			Ref.			Ref.			Ref.		
1-4	0.91	0.84-0.97		0.84	0.72-0.99		0.88	0.72-1.08		1.12	0.92-1.37		0.91	0.77-1.08	
≥5	0.90	0.79-1.03		0.95	0.73-1.24		0.94	0.67-1.33		0.82	0.53-1.25		0.63	0.43-0.92	
Missing teeth			<.0001			0.0001			<.0001			0.0003			0.0003
No	Ref.			Ref.			Ref.			Ref.			Ref.		
Yes	1.12	1.08-1.16		1.17	1.08-1.27		1.27	1.14-1.41		1.24	1.1-1.4		1.18	1.08-1.3	
Periodontitis			0.2751			0.0384			0.0035			0.0372			0.3083
No	Ref.			Ref.			Ref.			Ref.			Ref.		
Yes	1.02	0.99-1.05		1.08	1-1.16		0.87	0.79-0.95		1.12	1.01-1.25		1.04	0.96-1.13	
Comorbidity															
Obesity	1.03	1-1.07	0.0705	1.04	0.97-1.12	0.2588	0.75	0.68-0.84	<.0001	1.05	0.94-1.17	0.4163	1.13	1.04-1.22	0.0057
Hypertension	1.21	1.16-1.27	<.0001	1.10	1-1.21	0.0487	1.19	1.05-1.35	0.0064	1.06	0.92-1.22	0.4490	1.46	1.32-1.61	<.0001
Diabetes	1.28	1.21-1.36	<.0001	1.28	1.13-1.45	0.0001	1.20	1.01-1.42	0.0346	1.71	1.44-2.02	<.0001	1.20	1.034-1.38	0.0150
Heart disease	1.17	1.07-1.28	0.0006	0.95	0.77-1.16	0.5921	1.27	1-1.61	0.0511	0.96	0.70-1.32	0.8131	1.21	0.99-1.49	0.0637
Cerebrovascular disease	1.01	0.90-1.13	0.8742	1.06	0.83-1.35	0.6639	0.99	0.72-1.36	0.9592	0.97	0.66-1.43	0.8887	0.82	0.61-1.10	0.1898

	Head and Neck Cancer			Thyroid cancer			Bladder cancer			Pancreatic cancer			Kidney Cancer		
	OR	95% CI	*P*	OR	95% CI	*P*	OR	95% CI	*P*	OR	95% CI	*P*	OR	95% CI	*P*
Age group, y			<.0001			<.0001			<.0001			<.0001			0.0034
<65	Ref.			Ref.			Ref.			Ref.			Ref.		
≥65	3.47	2.92-4.12		0.23	0.18-0.3		4.53	3.65-5.62		2.62	2.04-3.37		1.56	1.16-2.1	
Sex			0.2621			<.0001			<.0001			0.15			<.0001
Male	Ref.			Ref.			Ref.			Ref.			Ref.		
Female	0.90	0.75-1.08		3.88	3.44-4.38		0.23	0.17-0.3		0.83	0.65-1.07		0.38	0.29-0.5	
Income			0.0003						0.1907			0.2946			0.4196
≤Third quantiles	Ref.			Ref.		<.0001	Ref.			Ref.			Ref.		
>Third quantiles	0.74	0.63-0.87		1.32	1.19-1.46		0.86	0.69-1.08		0.88	0.7-1.12		1.12	0.86-1.46	
Smoking			0.2244			0.0045			0.0364			0.2053			0.7509
Never	Ref.			Ref.			Ref.			Ref.			Ref.		
Ex-smoker	0.95	0.73-1.24		0.85	0.69-1.04		1.21	0.93-1.59		1.22	0.88-1.7		0.95	0.69-1.29	
Current smoker	1.15	0.95-1.40		0.77	0.65-0.9		1.32	1.07-1.62		1.25	0.97-1.62		1.06	0.84-1.35	
Alcohol consumption			<.0001			0.009			0.6164			0.8558			0.0978
<10 times per month	Ref.			Ref.			Ref.			Ref.			Ref.		
≥10 times per month	1.97	1.62-2.39		0.75	0.6-0.93		1.06	0.84-1.36		0.97	0.71-1.33		0.76	0.55-1.05	
Dental health status															
Dental caries			0.2814			0.0882			0.7593			0.3182			0.0280
0	Ref.			Ref.			Ref.			Ref.			Ref.		
1-4	0.99	0.73-1.35		0.81	0.67-0.99		1.10	0.77-1.58		0.83	0.53-1.29		0.51	0.29-0.89	
≥5	1.43	0.92-2.23		0.85	0.58-1.25		1.18	0.65-2.17		1.45	0.79-2.67		0.48	0.15-1.49	
Missing teeth			0.0006			<.0001			0.9114			0.0304			0.1657
No	Ref.			Ref.			Ref.			Ref.			Ref.		
Yes	1.32	1.13-1.55		0.78	0.7-0.88		1.01	0.83-1.24		1.27	1.02-1.58		0.84	0.66-1.07	
Periodontitis			0.6901			0.8246			0.7089			0.6893			0.3707
No	Ref.			Ref.			Ref.			Ref.			Ref.		
Yes	0.97	0.84-1.13		1.01	0.93-1.1		1.04	0.87-1.24		1.04	0.85-1.27		0.92	0.75-1.11	
Comorbidity															
Obesity	1.05	0.9-1.22	0.5383	1.13	1.03-1.23	0.0096	1.04	1.15	0.95-1.38	1.08	0.88-1.33	<.0001	1.44	1.18-1.75	0.0003
Hypertension	1.48	1.25-1.76	<.0001	1.07	0.95-1.2	0.2916	1.10	1.25	1.0-1.56	1.25	0.97-1.6	0.0064	1.87	1.49-2.35	<.0001
Diabetes	1.66	1.33-2.07	<.0001	0.95	0.79-1.15	0.6108	1.28	1.20	0.89-1.63	1.39	1.0-1.92	0.0346	1.57	1.16-2.14	0.0041
Heart disease	1.24	0.88-1.74	0.2252	1.05	0.81-1.37	0.7122	0.95	1.41	0.94-2.12	1.84	1.21-2.78	0.0511	1.65	1.09-2.51	0.0178
Cerebrovascular disease	1.69	1.18-2.4	0.0039	0.95	0.66-1.35	0.7531	1.06	1.32	0.81-2.17	0.79	0.39-0.6	0.9592	0.83	0.41-0.69	0.6138

	Gallbladder/biliary tract cancer			Esophageal Cancer			Hematologic malignancies		
	OR	95% CI	*P*	OR	95% CI	*P*	OR	95% CI	*P*
Age group, y			<.0001			<.0001			<.0001
<65	Ref.			Ref.			Ref.		
≥65	3.73	2.95-4.71		3.86	2.76-5.39		2.41	2.07-2.81	
Sex			0.1504			<.0001			0.009
Male	Ref.			Ref.					
Female	1.20	0.94-1.54		0.19	0.11-0.33		0.83	0.72-0.95	
Income			0.1003			0.0164			0.0087
≤Third quantiles	Ref.			Ref.			Ref.		
>Third quantiles	0.83	0.66-1.04		0.68	0.49-0.93		0.83	0.72-0.95	
Smoking			0.4794			0.0411			0.0123
Never	Ref.			Ref.			Ref.		
Ex-smoker	1.24	0.88-1.74		1.02	0.64-1.61		0.84	0.68-1.04	
Current smoker	1.09	0.83-1.43		1.47	1.06-2.02		0.79	0.67-0.93	
Alcohol consumption			0.001			<.0001			0.0074
<10 times per month	Ref.			Ref.			Ref.		
≥10 times per month	1.61	1.21-2.15		3.01	2.24-4.04		1.28	1.07-1.54	
Dental health status									
Dental caries			0.724			0.3428			0.9539
0	Ref.			Ref.			Ref.		
1-4	1.07	0.72-1.59		1.43	0.88-2.3		1.03	0.81-1.32	
≥5	0.75	0.33-1.68		1.13	0.46-2.77		0.96	0.6-1.54	
Missing teeth			0.0239			0.027			0.2256
No	Ref.			Ref.			Ref.		
Yes	1.28	1.03-1.6		1.40	1.04-1.88		0.92	0.8-1.06	
Periodontitis			0.1771			0.3849			0.7347
No	Ref.			Ref.			Ref.		
Yes	1.15	0.94-1.4		0.88	0.67-1.17		0.98	0.87-1.1	
Comorbidity									
Obesity	1.27	1.04-1.56	0.0189	0.60	0.43-0.84	0.0025	1.16	1.03-1.31	0.0140
Hypertension	0.78	0.59-1.02	0.0663	0.91	0.61-1.35	0.6401	1.28	1.11-1.49	0.0009
Diabetes	1.50	1.1-2.06	0.0112	1.21	0.73-2.0	0.4590	1.23	1.0-1.51	0.0514
Heart disease	1.28	0.78-2.1	0.3293	1.54	0.78-3.04	0.2176	1.10	0.81-1.5	0.5350
Cerebrovascular disease	0.91	0.47-1.78	0.7823	0.44	0.11-1.79	0.2504	1.17	0.81-1.68	0.3980

Adjusted for age, sex, income, smoking, alcohol consumption and comorbidities

*OR* Odd ratio, *CI* Confidence interval

In the information on dental health status, dental caries did not show significant associations with risk for all cancers. In addition, the number of dental caries was not related to cancer risk. However, missing teeth was a significant factor related to higher cancer risk. People with missing teeth showed a higher risk for all cancers (odds ratio (OR) 1.12 [95% confidence interval (CI), 1.08–1.16]. The risk was significantly higher, particularly in lung cancer, head and neck cancer, pancreatic cancer, liver cancer, biliary cancer, and esophageal cancer (OR 1.27 [95% CI, 1.14–1.41], 1.32 [95% CI, 1.13–1.55], 1.27 [95% CI, 1.02–1.58], 1.24 [95% CI, 1.1–1.4], 1.28 [95% CI, 1.03–1.6], 1.4 [95% CI, 1.04–1.88], respectively).

Because we adjusted for all other potential clinical or epidemiologic factors, missing teeth were confirmed as an independent factor impacting cancer risk, especially for some specific cancers. However, for thyroid cancer, missing teeth were associated with decreased cancer risk (OR 0.78 [95% CI, 0.7–0.88]. For periodontitis, it was not related to increased cancer risk in all cancers, but the risk of gastric and liver cancers increased with OR 1.08 [95% CI, 1.0–1.15] and 1.12 [95% CI, 1.01–1.25], respectively.

**Fig. 2:**
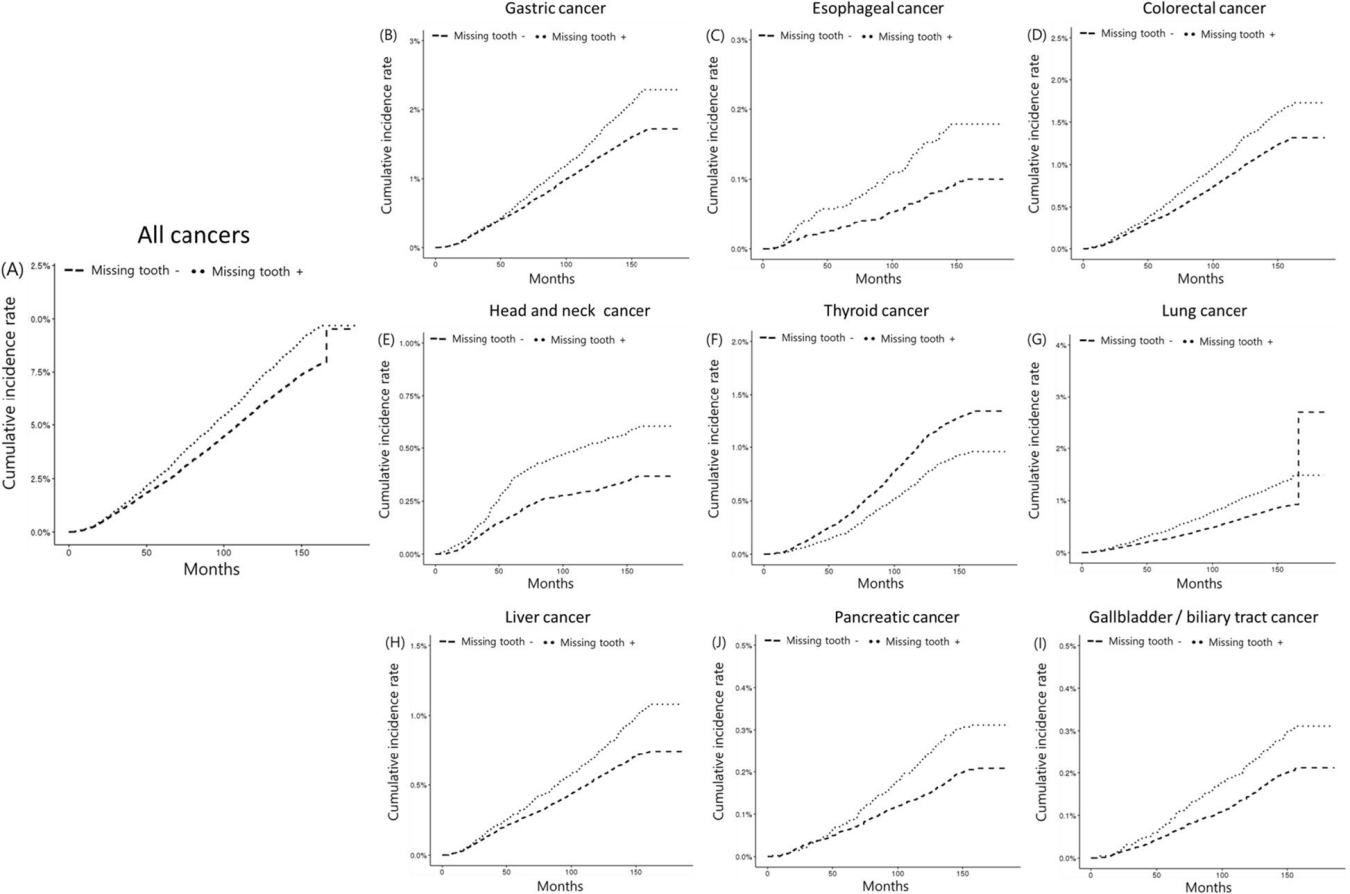
Cumulative incidence of several cancers according to the missing teeth

We analyzed the time to know how long it takes to diagnose cancer after the dental examination (Fig. 2). The median time to cancer diagnosis after the dental examination was 87 months (range, 51–119 months). With time, the incidence of all cancers increased, particularly the difference started from the beginning in the case of head and neck cancer.

## Discussion

In this study, from a large-scale Korean cohort who underwent a national health dental examination, we confirmed that missing teeth are the most important risk factor for several cancers among various dental health conditions. Compared to the group without cancer, the proportion of subjects with missing teeth in the group with cancer was significantly higher; however, only a slight difference was found for dental caries, and no difference was found for periodontitis. Also, 22.9% of the total population of this study had missing teeth, and 8.9% of the subjects with missing teeth were diagnosed with cancer after the dental examination. We identified that the population with missing teeth had an increased risk of all cancers, especially gastric, lung, liver, colorectal, head and neck, pancreatic, biliary, and esophageal cancers. Compared to other dental health indicators, including periodontitis or dental caries, missing teeth are objective and obvious clinical parameters. Therefore, missing teeth can be used as a reproducible and representative indicator of dental health status and cancer risk in the real world.

Several studies have examined the association between dental health status and various cancers. Most reported that missing teeth or periodontitis are associated with an increased risk of several cancers in diverse populations [6, 16–22]. In some studies, missing teeth showed significant associations with specific cancers such as lung, gastric, liver, esophageal, pancreatic, and head and neck cancers, which is consistent with the results of this study. In a study performed in Japan, missing teeth were associated with an increased risk of head and neck cancer (OR 1.68), esophageal cancer (OR 2.36), and lung cancer (OR 1.54) [19]. In a pooled analysis for assessing the risk of esophageal cancer, missing teeth were associated with an increased risk of esophageal cancer in Asians with OR 1.52 [17]. In addition, missing teeth were associated with increased lung cancer risk with a relative risk (RR) of 1.69 in a pooled analysis [18]. The risk for gastric cancer was increased with a hazard ratio (HR) of 1.54 in a prospective cohort study from Sweden and 1.65 in a Finnish cohort [23, 24]. For pancreatic cancer, the RR was 1.54 for missing teeth in a meta-analysis [21]. The risk was also increased for head and neck cancer with an RR of 2.0 in a meta-analysis [25].

This study showed no significant increase in the risk of bladder cancer, kidney cancer, or hematological malignancies with missing teeth. Studies on the association between missing teeth and these cancers have rarely been reported, and the results of only a few studies on the associations of these cancers with periodontal disease are controversial. In studies by Michaud et al. and Nwizu et al., periodontal disease was not associated with an increased risk of bladder or urinary tract cancers [26, 27]. For kidney cancer, there was an increased risk of periodontitis among male health professionals with an OR of 1.49, but there was no association in never-smokers in this population (OR 1.06) [26]. In addition, Michaud et al. reported that missing teeth were not associated with increased hematopoietic malignancy or lymphatic cancer risk in male health professionals [28].

To date, there is no precisely defined mechanism for the association between dental health and cancer, and many researchers have proposed several hypotheses. First, some researchers have suggested that inflammation caused by oral bacterial infection can promote systemic inflammation and systemic inflammatory cytokines, which play a role in initiating malignancies [29, 30]. Second, nitrosamine, a carcinogen produced by nitrate-reducing oral bacteria, has been reported as a triggering factor in gastrointestinal cancers [31, 32]. Third, chronic inflammation caused by oral bacteria can promote local inflammation in surrounding tissues [33, 34]. When we comprehensively review our results and those of previous studies, we can suggest that dental health status mainly affects the area of the head and neck, digestive tract, lung, liver, and biliary-pancreas where oral bacteria can reach. This phenomenon supports a mechanism of local inflammation and irritation of surrounding tissues by contact with oral bacteria, which can be the main cause of the increased risk of specific cancers rather than systemic stimulation to distant organs.

Indeed, numerous studies have identified some oral microbiota correlated with cancer risk. *Fusobacterium* spp., an oral bacterium, was detected in pancreatic cancer and *Fusobacterium*-positive patients with pancreatic cancer had a worse prognosis [35]. In addition, *Fusobacterium* species were found to be associated with increased colon cancer risk, and *Streptococcus* species were suggested to be influential in the development of lung cancer [36, 37]. *Porphyromonas gingivalis* and *Aggregatibacter actinomycetemcomitans* have also been identified as correlative species with an increased risk of pancreatic cancer [38]. There have been substantial reports on the correlation between specific oral microbiota and head and neck cancer [39].

Because increased risk has been reported, especially in some specific cancers, mucosal areas of the oral cavity or surrounding areas, including laryngopharyngeal, respiratory, and gastrointestinal regions, might be susceptible to cancer development. In a single-cell transcriptome assay of the human mucosa of participants with periodontitis and healthy participants, stromal and epithelial cells in the oral mucosa of periodontitis promoted inflammatory cell recruitment, and these cells actively participated in upregulating pathways related to cell adhesion, cytokine signaling, and biosynthesis. Moreover, stromal cells and epithelial cells in the oral mucosa increased cell-damage receptors in periodontitis distinctively [40]. This suggests that the oral mucosa has unique susceptibility to inflammation-associated diseases. Carcinogenesis is related to the inflammatory status under the complex interactions between host and immune factors; thus, cells in the oral mucosa and nearby structures can be directly affected by the inflammatory response triggered by the oral microbiota, consequently promoting cancer development. In addition, the transmission of oral microbiota to the biliary tract, pancreas, and the colorectal area is possible mainly by swallowing saliva, which may participate in the carcinogenesis of cancers in this area. However, oral microbiota can hardly reach the kidney, urinary tract, or hematopoietic organs. This could be the reason for the differences in the risk of cancer development in each organ.

Additionally, nutritional status can be suggested as the possible linker for the correlation between poor dental health status and cancer. Malnutrition or specific nutrient deficiency may influence maintaining healthy oral mucosa or cause destruction in dental enamel. Poor dental health status results in poor or unbalanced nutritional intake, making a vicious cycle. Moreover, nutritional imbalance also may affect the risk of incidence of several cancers. In previous studies, a high amount of sugar intake in the diet was reported to have a strong correlation not only with dental caries but also with the risk of various cancers[41, 42]. In addition, deficient or low folate status was reported to have an association with stomatitis caused by the reduced periodontal tissue's ability to protect it from bacterial irritants [43]. Folate deficiency also has an association with an increased risk of several cancers [44]. With regard to Vitamin D, accumulating studies have strongly suggested that Vitamin D deficiency has a negative effect on oral health causing defective tooth mineralization as well as increasing the risk of several cancers [45, 46]. Although we could not perform a related analysis on nutritional status because data on the nutritional status of the study subjects were not available, nutritional status can also be suggested to be a factor that can explain the association between poor dental health status and cancer.

In this study, missing teeth were associated with a lower risk of thyroid cancer. Risk analysis of thyroid cancer with missing teeth has rarely been conducted, and there is only one report in Japan on the risk of thyroid disease with missing teeth [19]. Also, there was no significant association with the risk of thyroid cancer in this study. However, the number of thyroid cancer cases was small, with only 121 of the 5,240 cancer patients included in the Japanese study; therefore, there might be a statistical concern. In addition, because only people with malignancy-suspected thyroid nodules take fine needle aspiration or biopsy, probably there are pathologically undiagnosed thyroid cancer cases. Therefore, the results of the study may not reflect the correlation between missing teeth and the real risk of thyroid cancer. However, during the follow-up period of the subjects included in this study, the incidence of thyroid cancer in Korea increased significantly to the extent that this was criticized for over-diagnosis [47]. In other words, it can be interpreted that the number of thyroid cancer patients in this presenting study reflects the actual incidence of thyroid cancer, not the downwardly estimated incidence. The reason subjects with missing teeth showed a significantly lower risk of thyroid cancer is difficult to explain fully; further research is needed to clarify this.

Interestingly, at the time of cancer diagnosis after dental examination, head and neck cancer already showed an increased incidence from the beginning of follow-up. Meanwhile, the incidence of other cancers gradually increased with time, according to the presence of missing teeth. This result might imply that poor dental health status, including missing teeth, is strongly associated with head and neck cancer, regardless of timing. At least for head and neck cancer, oral health status should not be regarded as a tool for the prediction of future cancer, it should be used as an indicator of an immediate and routine screening for head and neck cancer. Like the role of visual dental examinations detecting pre-malignant or malignant lesions in oral cavity for screening oral cavity cancer, a more impactful screening program for the inspection of the whole area for head and neck cancer is necessary especially for patients with poor dental health status.

Previously, several screening models for oral cancer have been proposed, but there is no definitive conclusion regarding the most effective model [48]. Because there is a lack of sufficient evidence regarding a reduction in oral cancer mortality, population-based oral screening using visual inspection is not recommended in most countries except Taiwan. In Taiwan, which has a similar national health screening system to Korea, biennial oral examinations were conducted for smokers and/or betel quid chewers. Cancer incidence was evaluated between screened and non-screened populations. In this analysis, screening effectively reduced both T-stage and mortality of head and neck cancer [49]. There have been no attempts to categorize high-risk groups based on dental health status. Thus, our results suggest that missing teeth should be considered an influential factor in defining high-risk groups for further evaluation of the effectiveness of dental screening in several cancers.

This study had some limitations. First, we could not obtain clinical information of individuals, including the reason for missing teeth, stage of cancer, treatment of cancer, and death, because of the characteristics of the data composition and the Personal Information Protection Act applied to the data by the government. Second, claims data did not include information on some cancers, including breast cancer, female and male genital tract cancers, and prostate cancer; therefore, analysis of these cancers could not be performed. For the analysis of these cancers, further investigation is planned with additional permission from the NHIS.

However, over 200,000 people living in Korea were included and followed up for a long time with qualified data provided by the public institution. Therefore, the results of this study have strong value as the results of the Korean nationwide data. Second, all dental health examinations were conducted by dentists in designated institutions for NHC. Therefore, the results of national dental health screening are trustworthy compared to other studies that used the self-questionnaire survey of participants.

In conclusion, Korean adults with missing teeth and dentists should be cautious about the higher risk of cancers, particularly head and neck, lung, gastrointestinal, hepatobiliary, and pancreatic cancers. In addition, it is necessary to develop a precise screening program for these cancers, particularly in populations with missing teeth.

## Data Availability

NHIS (National Health Insurance System) has been operating NHISS (National Health Insurance Sharing Service) to provide a sufficient support of policy and academic research by utilizing National Health information. When we use database from NHIS, first, IRB approval for the research subject in investigator’s institution is needed. After IRB approval, researchers have to inform their research subjects to NHISS website, then the research plan should be permitted. After permission by NHIS, researchers only can analyze data in “Data analysis room” located within the National Health Insurance Corporation in which PC for review and analysis of data is installed. Because the data are national and public material, exporting data is prohibited with legal restriction. Therefore, researchers can only get results from analysis after data analysis. In brief, data cannot be made publicly available or shared due to legal restrictions. Interested researchers may contact NHIS to request data access from the website “https://nhiss.nhis.or.kr/bd/ay/bdaya001iv.do ". Corresponding author can give an information on how to access data through NHISS.

## Abbreviations

NHC: National Health Checkups
NHIS: National Health Insurance System

## References

[1] Schwahn C, Polzer I, Haring R, Dorr M, Wallaschofski H, Kocher T, Mundt T, Holtfreter B, Samietz S, Volzke H (2013). Missing, unreplaced teeth and risk of all-cause and cardiovascular mortality. Int J Cardiol.

[2] Kim J, Kim HJ, Jeon J, Song TJ (2022). Association between oral health and cardiovascular outcomes in patients with hypertension: a nationwide cohort study. J Hypertens.

[3] Ingram K, Hayes MJ, Irving M, Wallace J (2021). What informs oral health and chronic disease policy development in Australia: a citation analysis. J Public Health Pol.

[4] Beheshti M, Badner V, Shah P, Margulis KS, Yeroshalmi F (2021). Association of Diabetes and Dental Caries Among US Adolescents in the NHANES Dataset. Pediatr Dent.

[5] Punceviciene E, Rovas A, Puriene A, Stuopelyte K, Vitkus D, Jarmalaite S, Butrimiene I (2021). Investigating the relationship between the severity of periodontitis and rheumatoid arthritis: a cross-sectional study. Clin Rheumatol.

[6] Michaud DS, Fu ZX, Shi J, Chung M (2017). Periodontal Disease, Tooth Loss, and Cancer Risk. Epidemiol Rev.

[7] Korea Disease Control and Prevention Agency, Public Health Weekly Report. 2022;13(9):489–92.

[8] Kim JW, Park JB, Yim HW, Lee J, Kwok SK, Ju JH, Kim WU, Park SH (2019). Rheumatoid arthritis is associated with early tooth loss: results from Korea National Health and Nutrition Examination Survey V to VI. Korean J Intern Med.

[9] Kononen E, Gursoy M, Gursoy UK. Periodontitis: A Multifaceted Disease of Tooth-Supporting Tissues. J Clin Med. 2019;8(8):1135. 10.3390/jcm8081135 PMC6723779 31370168

[10] Yamashita Y, Takeshita T (2017). The oral microbiome and human health. J Oral Sci.

[11] Crusz SM, Balkwill FR (2015). Inflammation and cancer: advances and new agents. Nat Rev Clin Oncol.

[12] Arthur JC, Gharaibeh RZ, Muhlbauer M, Perez-Chanona E, Uronis JM, McCafferty J, et al. Microbial genomic analysis reveals the essential role of inflammation in bacteria-induced colorectal cancer. Nat Commun. 2014;5:4724. 10.1038/ncomms5724 PMC4155410 25182170

[13] Costalonga M, Herzberg MC (2014). The oral microbiome and the immunobiology of periodontal disease and caries. Immunol Lett.

[14] National Health Insurance Sharing Service [https://nhiss.nhis.or.kr/bd/ab/bdaba000eng.do].

[15] Korea Disease Control and Prevention Agency, Manual for the dentists performing the National Dental Health Checkup. https://www.kdca.go.kr.

[16] Al-Maweri SA, Ibraheem WI, Al-Ak'hali MS, Shamala A, Halboub E, Alhajj MN (2021). Association of periodontitis and tooth loss with liver cancer: A systematic review. Crit Rev Oncol Hematol.

[17] Chen H, Nie SP, Zhu YH, Lu M. Teeth loss, teeth brushing and esophageal carcinoma: a systematic review and meta-analysis. Sci Rep-Uk. 2015;5:15203. 10.1038/srep15203 PMC4604458 26462879

[18] Chen Y, Zhu BL, Wu CC, Lin RF, Zhang X. Periodontal Disease and Tooth Loss Are Associated with Lung Cancer Risk. Biomed Res Int. 2020;2020:5107696. 10.1155/2020/5107696 PMC7403933 32802852

[19] Hiraki A, Matsuo K, Suzuki T, Kawase T, Tajima K (2008). Teeth loss and risk of cancer at 14 common sites in Japanese. Cancer Epidem Biomar.

[20] Lee K, Lee JS, Kim J, Lee H, Chang Y, Woo HG, Kim JW, Song TJ (2020). Oral health and gastrointestinal cancer: A nationwide cohort study. J Clin Periodontol.

[21] Maisonneuve P, Amar S, Lowenfels AB (2017). Periodontal disease, edentulism, and pancreatic cancer: a meta-analysis. Ann Oncol.

[22] Momen-Heravi F, Babic A, Tworoger SS, Zhang LB, Wu KN, Smith-Warner SA, Ogino S, Chan AT, Meyerhardt J, Giovannucci E (2017). Periodontal disease, tooth loss and colorectal cancer risk: Results from the Nurses' Health Study. Int J Cancer.

[23] Ndegwa N, Ploner A, Liu Z, Roosaar A, Axell T, Ye W (2018). Association between poor oral health and gastric cancer: A prospective cohort study. Int J Cancer.

[24] Abnet CC, Qiao YL, Dawsey SM, Dong ZW, Taylor PR, Mark SD (2005). Tooth loss is associated with increased risk of total death and death from upper gastrointestinal cancer, heart disease, and stroke in a Chinese population-based cohort. Int J Epidemiol.

[25] Wang RS, Hu XY, Gu WJ, Hu Z, Wei B (2013). Tooth loss and risk of head and neck cancer: a meta-analysis. PLoS ONE.

[26] Michaud DS, Liu Y, Meyer M, Giovannucci E, Joshipura K (2008). Periodontal disease, tooth loss, and cancer risk in male health professionals: a prospective cohort study. Lancet Oncol.

[27] Nwizu NN, Marshall JR, Moysich K, Genco RJ, Hovey KM, Mai XD, LaMonte MJ, Freudenheim JL, Wactawski-Wende J (2017). Periodontal Disease and Incident Cancer Risk among Postmenopausal Women: Results from the Women's Health Initiative Observational Cohort. Cancer Epidem Biomar.

[28] Michaud DS, Lu JY, Peacock-Villada AY, Barber JR, Joshu CE, Prizment AE, Beck JD, Offenbacher S, Platz EA (2018). Periodontal Disease Assessed Using Clinical Dental Measurements and Cancer Risk in the ARIC Study. Jnci-J Natl Cancer I.

[29] Hoare A, Soto C, Rojas-Celis V, Bravo D. Chronic Inflammation as a Link between Periodontitis and Carcinogenesis. Mediat Inflamm. 2019;2019:1029857. 10.1155/2019/1029857 PMC6458883 31049022

[30] Landskron G, De la Fuente M, Thuwajit P, Thuwajit C, Hermoso MA (2014). Chronic inflammation and cytokines in the tumor microenvironment. J Immunol Res.

[31] Nair J, Ohshima H, Nair UJ, Bartsch H (1996). Endogenous formation of nitrosamines and oxidative DNA-damaging agents in tobacco users. Crit Rev Toxicol.

[32] Meyer MS, Joshipura K, Giovannucci E, Michaud DS (2008). A review of the relationship between tooth loss, periodontal disease, and cancer. Cancer Cause Control.

[33] Kageyama S, Takeshita T, Takeuchi K, Asakawa M, Matsumi R, Furuta M, Shibata Y, Nagai K, Ikebe M, Morita M, et al. Characteristics of the Salivary Microbiota in Patients With Various Digestive Tract Cancers. Front Microbiol. 2019;10:1780:104710. 10.3389/fmicb.2019.01780 PMC6688131 31428073

[34] Li YC, Tan XX, Zhao XD, Xu ZF, Dai W, Duan WY, Huang SH, Zhang EJ, Liu JC, Zhang SW et al: Composition and function of oral microbiota between gingival squamous cell carcinoma and periodontitis. Oral Oncol 2020, 107. 10.1016/j.oraloncology.2020.104710 32371264

[35] Mitsuhashi K, Nosho K, Sukawa Y, Matsunaga Y, Ito M, Kurihara H, Kanno S, Igarashi H, Naito T, Adachi Y (2015). Association of Fusobacterium species in pancreatic cancer tissues with molecular features and prognosis. Oncotarget.

[36] Flanagan L, Schmid J, Ebert M, Soucek P, Kunicka T, Liska V, Bruha J, Neary P, Dezeeuw N, Tommasino M (2014). Fusobacterium nucleatum associates with stages of colorectal neoplasia development, colorectal cancer and disease outcome. Eur J Clin Microbiol.

[37] Zhang WQ, Luo JW, Dong XP, Zhao SK, Hao YT, Peng CL, Shi HB, Zhou Y, Shan L, Sun QF (2019). Salivary Microbial Dysbiosis is Associated with Systemic Inflammatory Markers and Predicted Oral Metabolites in Non-Small Cell Lung Cancer Patients. J Cancer.

[38] Stasiewicz M, Kwasniewski M, Karpinski TM. Microbial Associations with Pancreatic Cancer: A New Frontier in Biomarkers. Cancers. 2021;13(15):3784. 10.3390/cancers13153784 PMC8345173 34359685

[39] Zhang L, Liu Y, Zheng HJ, Zhang CP. The Oral Microbiota May Have Influence on Oral Cancer. Front Cell Infect Mi. 2020;9:476. 10.3389/fcimb.2019.00476 PMC6974454 32010645

[40] Williams DW, Greenwell-Wild T, Brenchley L, Dutzan N, Overmiller A, Sawaya AP, Webb S, Martin D, Genomics NN, Computational Biology C, et al. Human oral mucosa cell atlas reveals a stromal-neutrophil axis regulating tissue immunity. Cell. 2021;184(15):4090–104. 10.1016/j.cell.2021.05.013 PMC8359928 34129837

[41] Rugg-Gunn AJ (1993). Nutrition, diet and dental public health. Community Dent Health.

[42] Debras C, Chazelas E, Srour B, Kesse-Guyot E, Julia C, Zelek L, Agaesse C, Druesne-Pecollo N, Galan P, Hercberg S (2020). Total and added sugar intakes, sugar types, and cancer risk: results from the prospective NutriNet-Sante cohort. Am J Clin Nutr.

[43] Waerhaug J (1967). Prevalence of periodontal disease in Ceylon. Association with age, sex, oral hygiene, socio-economic factors, vitamin deficiencies, malnutrition, betel and tobacco consumption and ethnic group. Final report. Acta Odontol Scand.

[44] Pieroth R, Paver S, Day S, Lammersfeld C (2018). Folate and Its Impact on Cancer Risk. Curr Nutr Rep.

[45] Botelho J, Machado V, Proenca L, Delgado AS, Mendes JJ. Vitamin D Deficiency and Oral Health: A Comprehensive Review. Nutrients. 2020;12(5):1471. 10.3390/nu12051471 PMC7285165 32438644

[46] Feldman D, Krishnan AV, Swami S, Giovannucci E, Feldman BJ (2014). The role of vitamin D in reducing cancer risk and progression. Nat Rev Cancer.

[47] Ahn HS, Kim HJ, Welch HG (2014). Korea's thyroid-cancer "epidemic"–screening and overdiagnosis. N Engl J Med.

[48] Warnakulasuriya S, Kerr AR (2021). Oral Cancer Screening: Past, Present, and Future. J Dent Res.

[49] Chuang SL, Su WW, Chen SL, Yen AM, Wang CP, Fann JC, Chiu SY, Lee YC, Chiu HM, Chang DC (2017). Population-based screening program for reducing oral cancer mortality in 2,334,299 Taiwanese cigarette smokers and/or betel quid chewers. Cancer.

